# Expert consensus on the long-term use of lanadelumab in hereditary angioedema: Toward harmonized care 

**DOI:** 10.5414/ALX02622E

**Published:** 2026-05-05

**Authors:** Emel Aygören-Pürsün, Jens Greve, Inmaculada Martinez-Saguer, Susanne Trainotti, Mathias Sulk, Bettina Wedi, Ellen Witte-Händel, Markus Magerl

**Affiliations:** 1 University Hospital Frankfurt, Goethe University, Frankfurt,; 2 Department of Otorhinolaryngology, Head and Neck Surgery, Ulm University Medical Center, Ulm,; 3 Haemophilia Centre Rhine Main, Frankfurt/Main,; 4 Technical University of Munich, TUM School of Medicine and Health, Department of Otorhinolaryngology, Head and Neck Surgery, TUM University Hospital, Munich,; 5 Department of Dermatology, University of Münster, Münster,; 6 Department of Dermatology and Allergy, Comprehensive Allergy Center, Hannover Medical School, Hannover,; 7 Angioedema Center of Reference and Excellence (ACARE), Institute of Allergology, Charité – Universitätsmedizin Berlin, Corporate Member of Freie Universität Berlin and Humboldt-Universität zu Berlin,; 8 Global Allergy and Asthma Excellence Network, ACARE/UCARE coordinating office, and; 9 Fraunhofer Institute for Translational Medicine and Pharmacology ITMP, Immunology and Allergology, Berlin, Germany

**Keywords:** angioedema, consensus statements, HAE, hereditary angioedema, Lanadelumab, long-term prophylaxis

## Abstract

Background: Hereditary angioedema (HAE) is a rare, potentially life-threatening disease caused in most cases by C1 inhibitor deficiency. Lanadelumab, a monoclonal antibody targeting plasma kallikrein, is an effective long-term prophylactic (LTP) treatment for HAE. However, consensus on best practices remains lacking. Objectives: This study aimed to report consensus statements on key principles on long-term lanadelumab therapy for HAE in Germany developed at an HAE LTP Expert Meeting in the year 2024 in Frankfurt, Germany. Materials and methods: A multidisciplinary panel of seven German HAE experts participated in a consensus process to align current guidelines with real-world clinical practice. Following literature review and debate, keynotes were drafted, refined, and voted on. Consensus was defined as ≥ 70% agreement. Key domains included: shared decision-making; flexibility in initiating or adjusting prophylaxis; structured patient education; self-administration; individualized dosing; emergency medication availability; and proactive follow-up, including specific guidance for women of childbearing age. Results: Ten core consensus statements were developed, achieving unanimous (100%, n = 9/10 statements) or strong (≥ 85%, n = 1/10 statements) agreement. It is recommended that the decision to initiate long-term prophylaxis be made through shared decision-making and that the decision made can and should be adjusted again in the further course of treatment. It is advisable to train patients in the technique of self-injection and to start therapy with lanadelumab with a 2-week injection interval in accordance with the product information. The injection interval should be adjusted to the individual patient, and all well-controlled patients should be offered the option of extending the interval without compromising the goal of complete disease control. Even and especially when the prophylaxis is well tolerated, emergency medication must not be neglected. Conclusion: These consensus statements provide a practical, expert-endorsed framework for implementing lanadelumab LTP in clinical practice emphasizing individualized treatment aligned with international guidelines and patient needs.

## Introduction 

Hereditary angioedema (HAE) is a rare genetic disease characterized by recurrent attacks of swelling. Depending on the location of the swelling, these cause temporary disfigurement, functional limitations of the limbs, pain, or life-threatening respiratory distress. The symptoms themselves, but also their frequency and unpredictability of occurrence, lead to a massive restriction of lifestyle and quality of life for many patients [1]. The effects of the swelling attacks can be reduced through the early application of needs-oriented therapy, which allows a certain degree of disease control [2]. In many cases, complete disease control and the desired normalization of life as the recommended treatment goals can only be achieved through the use of long-term prophylaxis (LTP). In recent years, the number of options available for LTP has multiplied, allowing individualized treatment plans to be implemented [3]. There is a broad and long-standing consensus in the treatment community for the individualization of therapy for HAE [4]. Both the pronounced variability of disease activity as well as large differences in response to different therapies and the compatibility of therapy application with different lifestyles make individualization sensible and necessary [5]. 

Lanadelumab is a monoclonal antibody directed against plasma kallikrein and has been approved for the LTP of HAE since 2018 [6]. It is licensed for subcutaneous administration at a dose of 300 mg every 2 weeks in the beginning (dosing for children under 12 years of age differs) with extension of injection intervals to 4 weeks in symptom-free patients. The international guideline recommends the use of lanadelumab (alongside berotralstat and C1 inhibitor (C1INH)) as a first choice for LTP [7]. The pivotal studies leading to approval demonstrated that lanadelumab is efficacious in preventing angioedema attacks in HAE-C1INH patients aged 2 years and older and indicated a bland safety profile. Open-label extension studies clearly confirm these results [8]. 

Recently, a paper was published on an observational study in Germany, Austria, France, and Greece on the use of lanadelumab in patients with HAE, in which, in addition to various efficacy parameters, the real-life use was described in detail [9]. The study showed that the option to individualize dosing provided by the 2- or 4-week administration specified in the prescribing information is implemented in the real world to a much greater extent than expected. Just 6 months after initiation of treatment with lanadelumab, almost 30% of patients extended the intervals beyond the initial 14 days. This proportion rose to over 73% in the further course (36 months), in Germany even to 80%. Between 6.6 and 19.8% of patients used intervals longer than 28 days (Germany between 5 and 30%), which strictly speaking corresponds to off-label use. Despite the extension of the dosing intervals, the efficacy measured by the attack-free rate (AFR) (proportion of patients who had zero HAE attacks) per month remained high (postindex interval AFR range: Q2W, 61.2%; Q3W-like, 77.1%; Q4W, 76.2%; Q5W-like, 88.8%; > Q6W, 92.8%). 

The aim of the HAE LTP expert meeting described here was to facilitate exchange, try to align different approaches applied in Germany, and to potentially formulate recommendations for the initiation and implementation of LTP, particularly with lanadelumab, which take into account the current data, reflect the reality of use and go beyond the general recommendations of the international guideline. 

## Materials and methods 

### Meeting expert panel and objectives 

A multidisciplinary group of 7 German HAE experts (Table 1), including dermatologists, pediatricians, internists, and otolaryngologists was convened for the HAE LTP Expert Meeting in Frankfurt/Main, Germany on October 23, 2024, with an alignment mission regarding the long-term treatment of HAE patients with lanadelumab. The experts participated in person at the meeting with the aim of analyzing the treatment landscape concerning current real-world use of lanadelumab LTP in clinical practice in Germany and to define the key points of best practice in HAE management. At the opening of the meeting, the goals were introduced, which had been developed in the preparation stage of the meeting in an open discussion. All meeting attendees were involved in the definition of the meeting objectives and in the consensus process. 

### Development of consensus statements 

Following the meeting agenda, the consensus-building process covered 3 main modules to be addressed: 

First, in an initial exchange session, all participants contributed to the general debate of the clinical experience with lanadelumab in the LTP of HAE attacks and in light of the respective literature. 

Second, consensus statement forming was conducted as a multi-stage, feedback-based re-iterative process of drafting, discussing, and refining. To initiate this process, experts were split and assigned to one of 3 consensus groups, which were defined based on a specific subtopic in the field of LTP of HAE. Pre-defined subtopics included i) introduction, ii) initiation, and iii) management of the implementation of lanadelumab at individual centers in Germany. Each working group consisted of 2 or more experts (Table 1). 

In detail, consensus statements were developed using a method that included the following consecutive steps. In the topic-based group-setting, experts proposed a series of keynotes based on the group discussions and literature review. Group work related to the respective specific subject included exchange on state-of-the-art treatment and experience in their daily clinical practice. Keynotes to be considered were summarized, discussed, and developed into a concept indicating current controversy, precautions, challenges, and needs. 

The results of the group work were presented in a plenary session to all participants. A total of 20 keynotes were presented to all panelists and edited for duplicates followed by two rounds of discussion, debate, and revision held with all delegates. Following the development process, final selection and agreement on the most important points including all group opinions culminated in drafting of statements. In repeated rounds of review and refinement, all participants had the opportunity to evaluate the statements and provide feedback. A total of 10 statements were submitted for voting. The consensus was determined in a general vote by a show of hands. Consensus level with predefined agreement level (Table 2) established by the entire meeting group via simple voting was assessed by counting of all votes (Table 3). Accordingly, satisfactory consensus was defined as 70% (n = 5/7) agreement. Strong consensus was defined as 85% (n = 6/7) agreement. Opposing votes were documented and discussed. Statements with less than 70% agreement were regarded as no consensus, respectively. The consensus meeting was conducted and the consensus statements were developed in German. For this publication, the consensus statements were carefully translated into English. The accuracy of this translation was reviewed and approved by all authors. The original German-language consensus statements are provided in the supplement. Finally, in the subsequent third session, future steps were evaluated. 

## Results 

### Meeting objectives 

Prior to the meeting, all participating experts reached full consensus on the 3 overarching objectives guiding the meeting. These included: i) describing and comparing how the introduction, initiation, and monitoring of LTP treatment with lanadelumab on the patient level are implemented across different treatment centers in Germany; ii) identifying and defining key points of expert consensus; and iii) developing strategies to implement this consensus within the German HAE treatment landscape. 

### Workshop results 

**Figure 1:**
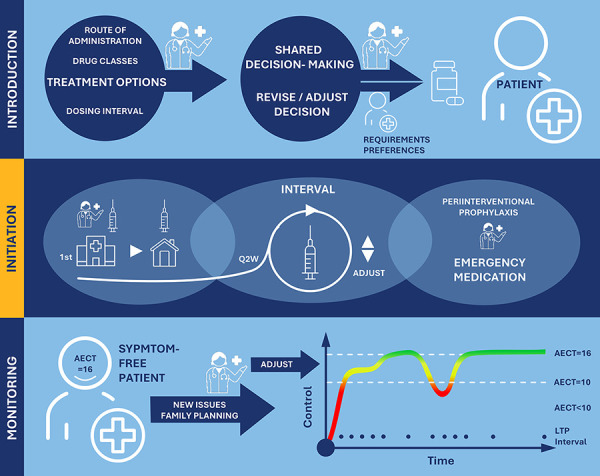
Three-step practical guideline for the long-term treatment of hereditary angioedema patients with lanadelumab in Germany.

During the group work phase, each of the 3 expert working groups, focusing on introduction (1), initiation (2), and management (3) of LTP treatment of HAE patients with lanadelumab, identified a series of keynotes (Table 3). These were based on the group experts’ clinical experience, literature review, and structured discussion and reflected both shared practices and current challenges across the participating experts’ affiliated centers in Germany. Identified keynotes were categorized into one of the following domains: shared decision-making (SDM) and patient education, practical aspects of therapy initiation and switch, clinical monitoring and outcome assessment, communication, and follow-up strategies. These provided the basis for the subsequent drafting of consensus statements that were developed into a 3-step practical guideline (Figure 1). 

### Consensus statements for the long-term treatment of HAE patients with lanadelumab 

Of the 10 final core statements developed in the plenary session, 9 achieved unanimous consensus (agree 7/7, 100%), and 1 was supported with a strong consensus (agree, 6/7, 85.7%) (Table 4). Voting results supported by relevant literature are presented in detail below. 

### Consensus statements on the introduction of lanadelumab long-term prophylaxis in hereditary angioedema: 

**1) The decision to initiate long-term prophylaxis is made through a shared decision-making process.**

**Agree 7/7, 100%**

Discussions of therapeutic options are inherently influenced by prior preferences and clinical history, and a transparent dialogue ensures informed choice. SDM has become a standard in chronic disease management and is particularly relevant in rare diseases such as HAE, where patient burden, treatment goals, and individual circumstances may vary significantly and where adherence to agreed treatment plans is crucial for the treatment outcome. Clinical guidelines endorse SDM as a key principle in determining the need for LTP, especially considering that attack frequency, severity, and impact on quality of life differ widely among patients [10, 11]. Moreover, studies emphasize that involving patients in therapeutic decisions improves adherence and satisfaction while fostering trust between patient and clinician [12]. In the context of LTP for HAE, SDM facilitates a balanced discussion of risks and benefits of treatment options leading to improved clinical outcomes and quality of life. This patient-centered approach supports individualized care and is recommended by recent international guidelines [7]. 

**2) It is emphasized that the decision for long-term prophylaxis is not final and can be revised or adjusted at any time.**

**Agree 7/7, 100%**

The variable disease activity necessitates flexibility in therapeutic decisions. Patients may transition between prophylaxis and on-demand regimens based on disease control, lifestyle changes, or life stage-associated characteristics such as pregnancy. Studies show that individualizing therapy over time improves adherence and patient satisfaction, highlighting the importance of a dynamic and revisable treatment approach [5, 13]. Evidence supports that LTP treatment plans should be continuously reviewed and adapted based on patient response, changes in disease severity, or lifestyle [11]. This approach allows for escalation, de-escalation, or switching of prophylactic agents without compromising safety or efficacy [8]. 

**3) Following an overview of the approved therapeutic options, including mode of administration and dosing intervals, the treatment choices are presented in detail, taking into account individual patient factors and preferences.**

**Agree 7/7, 100%**

A comprehensive understanding of therapeutic modalities is essential for informed decision-making. Clinical studies confirm that route and frequency of administration are major drivers of treatment choice [14]. Counseling on available LTP options, discussing differences in administration routes, dosing frequency, and safety profiles, tailored to each patient’s lifestyle and preferences, aligns with real-world patient satisfaction and adherence outcomes [15]. Presenting these aspects alongside efficacy and safety data enables patients and clinicians to select the most suitable regimen, particularly considering factors such as needle phobia and comorbidities. Neutral, comparative educational materials further empower patients during this formative stage. 

### Consensus statements on the initiation of lanadelumab long-term prophylaxis in hereditary angioedema: 

**4) After appropriate training in the injection technique, the administration of lanadelumab can be carried out by the patient. All patients should be offered the opportunity to receive their first injection at the treatment center.**

**Agree 6/7, 85.7%**

Self-administration of lanadelumab has been shown to be safe, effective, and well tolerated [8]. Training programs improve patient confidence and technique, reducing administration errors and adverse events. Providing the first injection under medical supervision allows clinicians to monitor for hypersensitivity reactions and ensure correct technique [16]. This practice aligns with the ongoing trend toward home-based therapies in chronic disease management [17]. The reason this statement did not achieve unanimous agreement is that 1 participant disagreed with the wording “All patients should be offered the opportunity to receive their first injection in the treatment center,” as this leaves open the possibility of the patients receiving the first injection outside the prescribing center or even administering it themselves. The participant who voted “no” believes that the first injection should only be administered in the HAE center under medical supervision. The remaining 6 participants support the wording of the option for a first injection outside the HAE center, but agree that a first injection in the center would be preferable and should definitely be offered. 

**5) Treatment with lanadelumab in adolescent and adult patients is started with a biweekly injection interval and subsequently individualized based on patient response.**

**Agree 7/7, 100%**

Data from clinical trials support initiating lanadelumab at a dose every 2 weeks, showing that interval adjustments are feasible depending on patient response. Individualization of dosing intervals, based on symptom control and tolerability, is supported by real-world data demonstrating that many patients maintain efficacy with extended intervals up to 4 weeks [18]. This flexibility helps optimize treatment burden while maintaining disease control. Maintaining an open dialogue ensures that patients feel empowered to report concerns and modify treatment as needed, improving long-term disease control and minimizing overtreatment [3, 11]. 

**6) Additional therapies**

**a) Emergency medication must continue to be readily available and carried by the patient, even in the absence of attacks.**

**Agree 7/7, 100%**

**b) Peri-interventional prophylaxis should be considered in consultation with the relevant medical specialties.**

**Agree 7/7, 100%**

Current guidelines recommend maintaining rescue medication alongside prophylaxis as a standard of care [7]. There is the clinical observation that patients with long attack free intervals tend to “forget” to carry their emergency medication with them. However, as no LTP is 100% efficient, the absence of attacks cannot be guaranteed. Therefore, also well-controlled patients need to be reminded regularly to continue being vigilant and to carry their emergency medication with them. Despite effective prophylaxis with lanadelumab, breakthrough attacks can occur [19]. Moreover, peri-interventional prophylaxis remains a critical consideration [20]. 

### Statements on monitoring during lanadelumab long-term prophylaxis use in hereditary angioedema: 

**7) In most patients, extension of the injection interval is feasible without loss of efficacy and may be offered to asymptomatic patients.**

Emerging evidence supports the feasibility of extending lanadelumab injection intervals beyond the biweekly schedule in asymptomatic patients [18]. In fact, long-term extension studies and registry data indicate maintained disease control for intervals within (up to 28 days) or outside (more than 28 days, i.e. off-label use) the recommended treatment regimen, reducing treatment burden and improving quality of life [16]. In fact, results from the INTEGRATED study involving 198 HAE type I/II patients show a rapid and sustained improvement in attack-free rates, increasing from 0% pre-treatment to 54.4% at 12 months. Moreover, 72.7% of patients were able to extend dosing intervals with maintained efficacy, complementing clinical trial data [9]. This strategy requires close monitoring to promptly identify any recurrence of attacks. 

**8) Adjustment of the injection interval during treatment must always align with the primary therapeutic goal of complete disease control.**

**Agree 7/7, 100%**

The principal objective of long-term HAE management is the complete disease control and normalization of life [7]. Adjustments to dosing intervals should be made cautiously, prioritizing complete disease control and absence of symptoms. Any economic benefit of interval extension should be regarded as a byproduct but must not be the goal of the interval extension [21]. Treatment decisions must carefully balance minimizing burden of treatment and achieving complete disease control. 

**9) Patients should be encouraged to proactively report any relevant changes (e.g., comorbidities, changes in life circumstances, or disease activity) to the treatment center. This does not affect the relevance of regular follow-up visits.**

**Agree 7/7, 100%**

Proactive patient communication enhances early detection of disease progression, treatment-related complications, or comorbidity changes. Patient engagement in reporting new symptoms or life changes facilitates timely therapeutic adjustments and optimizing outcomes [11]. Nevertheless, regular follow-up remains critical to comprehensive assessment, including laboratory monitoring and outcome evaluation tools such as the Angioedema Control Test (AECT) [22] and the Angioedema Quality of Life Questionnaire (AE-QoL) [23]. 

**10) In women of childbearing age, family planning and reproductive intentions should be discussed and considered both prior to and during treatment.**

**Agree 7/7, 100%**

Women with HAE in their reproductive years require tailored counseling regarding the implications of prophylactic treatments on pregnancy and breastfeeding [24, 25]. While lanadelumab’s safety profile during pregnancy remains under investigation, current expert opinion advocates for individualized risk-benefit analysis and close monitoring [26]. During the development of lanadelumab, there were no indications of any harmful effects on the fetus, and there have been no reports of such effects in the literature since its approval. The German product information contains the following warning: “As a precautionary measure, the use of lanadelumab during pregnancy should be avoided”. Although it is known that IgG1 immunoglobulins do not cross the placenta in the first trimester, there is currently no clear recommendation as to whether the use of lanadelumab should be paused during the period of planning to conceive or only after pregnancy has been confirmed. Family planning discussions enable informed decisions and coordination with specialists, ensuring optimal maternal and fetal outcomes. This holistic approach aligns with best practices for managing chronic diseases in women of childbearing potential. 

## Discussion 

This expert consensus provides a practice-oriented approach to LTP with focus on lanadelumab in HAE care in Germany (Figure 1). Reflecting the collective experience of leading HAE experts, the ten statements address key elements of introduction to and initiation of LTP, as well as long-term management. The high level of agreement reached underscores the relevance and applicability of these recommendations across clinical settings. 

One key point that emerged from the consensus process is the importance of SDM in initiating LTP. HAE, as a rare and heterogenous disease, necessitates individualized therapeutic approaches. The variability in attack frequency, severity, and impact on quality of life requires treatment plans that are not only evidence-based but also align with patient life and preferences. By advocating for SDM as the starting point of LTP discussions, the consensus aligns with both international HAE guidelines and broader chronic disease management strategies. Moreover, the consensus emphasizes the dynamic nature of treatment decisions, highlighting that therapeutic plans can and should be revisited based on changes in disease activity or life circumstances. This flexibility is essential in real-world care and is increasingly seen as a hallmark of patient-centered management. 

The practical considerations regarding the initiation of LTP reflect current standards and address real-world implementation barriers. The conditional recommendation to administer the first dose of lanadelumab in a supervised setting while promoting subsequent self-administration recognizes both safety and confidence. This approach aligns with practices of other biologic therapies across chronic diseases, where home administration supported by training is linked with improved adherence and reduced healthcare utilization. Furthermore, the consensus emphasizes the importance of reminding patients, especially those who have been symptom-free for a long time, of the need to carry emergency medication. The authors´ observations show that some long-term symptom-free patients tend to become negligent in this regard. 

Importantly, the consensus affirms that lanadelumab treatment should begin at a biweekly interval, in line with the pivotal HELP and HELP OLE study protocols but also supports interval extension based on individual patient response [8]. Recent real-world data and long-term observational studies suggest that extending injection intervals may reduce the number of injection days (and thereby the burden of treatment) without compromising the overall efficacy of lanadelumab [16, 18, 27]. The consensus reflects this evidence while cautioning that any such adjustment must be guided by disease control rather than convenience or cost effectiveness. The balance between optimizing treatment burden and maintaining complete symptom control characterizes the broader ethical standard of the recommendations, that is to support flexibility without compromising safety or efficacy. Robust monitoring remains a cornerstone of effective long-term management. The use of validated outcome measures such as the AECT and AE-QoL complements clinical and personal information to individualize the treatment. These tools not only facilitate objective evaluation of treatment efficacy but also foster shared understanding between clinicians and patients. In parallel, patient-reported information – such as changes in comorbidities, lifestyle, or disease activity – is considered equally important. Encouraging patients to proactively engage with their treatment center ensures timely adaptations. 

Special attention was given to reproductive health considerations for women of childbearing age. The consensus appropriately emphasizes the importance of preemptive discussions around pregnancy and breastfeeding. Given the limited but evolving safety data on lanadelumab use during pregnancy, individualized counseling and multidisciplinary collaboration are critical. This proactive approach aligns with ethical and clinical standards in reproductive-age chronic disease management and reflects a commitment to holistic patient care. 

Despite the strengths of consensus reached for all statements, several limitations need to be discussed. First, while the expert panel was composed of highly experienced HAE specialists, the size of the group was small, and all members were based in Germany. As such, findings may only reflect regional practice or perspectives. Second, although informed by current literature, the consensus was not based on a formal systematic review, and some emerging data may not have been included. Lastly, the voting process relied on show-of-hands agreement, which does not capture the nuances of individual disagreements or conditional agreement. 

Future steps may include real-world validation of these consensus statements involving evaluation of clinical outcomes related to interval extension, monitoring, and patient satisfaction. In addition, future expert meetings should potentially consider incorporating broader input, to refine recommendations further. As the landscape of HAE therapeutics continues to evolve, particularly with emerging prophylactic agents, ongoing evaluation and adaptation of guidance will be essential. 

In conclusion, this consensus statement provides a practical, evidence-aligned framework for implementing LTP with lanadelumab in HAE patients in Germany. Through an emphasis on SDM, flexible yet controlled initiation and monitoring strategies, as well as proactive patient engagement, the recommendations support a high standard of individualized care. Its use in clinical practice promises better results, optimized resource utilization, and improved quality of life for patients with HAE. 

## Funding and acknowledgment 

Initial discussions which subsequently formed the basis of this article occurred during a face-to-face meeting funded by Takeda Pharma Vertrieb GmbH & Co. KG, where individuals were compensated for their time according to fair market value rates. The authors want to thank Takeda for organizing this meeting. The decision to develop a manuscript expanding on these discussions was made solely by the author group, with no direction, influence, or funding from Takeda. Takeda reviewed a final version of the manuscript for medical accuracy only prior to submission. The views and opinions contained within this article are those of the authors only and do not necessarily represent the views of Takeda. 

## Conflict of interest 

BW reports personal honoraria for lectures, presentations, or educational events from ALK-Abéllo, Bencard, CSL Behring, Novartis, Takeda and support for attending meetings and/or travel from CSL Behring, Novartis, Takeda, and participation on one-day advisory boards from Biocryst, CSL Behring, Kalvista, Novartis, Sanofi-Aventis, Takeda. 

EAP has received advisor, consultant, speaker fees, and/or grants from Astria, Biocryst, CSL Behring, Intellia, Kalvista, Otsuka, Pharvaris, and Takeda. 

IMS reports personal honoraria for lectures, presentations, or educational events from CSL Behring, Biocryst, Takeda, KalVista and support for attending meetings and/or travel from CSL Behring, Biocryst, Takeda, KalVista and participation on one-day advisory boards from CSL Behring, Biocryst, KalVista, Pharvaris, Takeda and sponsoring for dedicated patient events from BioCryst, CSL Behring, KalVista, Takeda. 

JG reports personal honoraria for lectures, presentations or educational events from CSL Behring, Biocryst, Takeda and support for attending meetings and/or travel from CSL Behring, Biocryst, Takeda, and participation on one-day advisory boards from CSL Behring, Biocryst, KalVista, Otsuka Pharma, Takeda and sponsoring for dedicated patient events from BioCryst, CSL Behring, KalVista, Otsuka Pharma, Takeda. 

MM has received speaker/advisor fees and/or research funding and/or was investigator in clinical trials from/of Argo, Astria, BioCryst, CSL Behring, Ionis/Otsuka, Intellia, KalVista, Pharvaris, and Takeda. 

MS reports personal honoraria for lectures, presentations, or educational events from Ärzteverband deutscher Allergologen, Ärztekammer Westfalen Lippe, AstraZeneca, Bencard, BioCryst, Blueprint Medicines, CSL Behring, Kalvista, HAL Allergie, Healthcare Deutschland, LEO Pharma, Novartis, RG Gesellschaft für Information und Organisation, Sanofi, Takeda, Unna Akademie and support for attending meetings and/or travel from Biocryst, CSL Behring and participation on one-day advisory boards from AstraZeneca, BioCryst, CSL Behring, LEO Pharma, Novartis, Sanofi, Takeda. 

ST reports personal honoraria for lectures, presentations, or educational events from CSL Behring, Otsuka Pharma, Takeda and support for attending meetings and/or travel from CSL Behring, Ionis Pharmaceuticals, Takeda, and participation on one-day advisory boards from CSL Behring, Takeda, Otsuka Pharma, and sponsoring for dedicated patient events from BioCryst, CSL Behring, KalVista, Otsuka Pharma, Takeda. 

**Table 1.** HAE Expert Consensus Symposium 2024 panel. The panelists, respective affiliations, and assigned consensus group. Consensus groups are numbered according to which area of long-term prophylactic treatment is covered: introduction (1), initiation (2), management (3).

**Meeting expert panel**			
**First name**	**Last name**	**Affiliation**	**Consensus group**
Emel	Aygören-Pürsün	University Hospital Frankfurt, Goethe University, Frankfurt, Germany.	1
Jens	Greve	Department of Otorhinolaryngology, Head and Neck Surgery, Ulm University Medical Center, Ulm, Germany.	1
Markus	Magerl	Urticaria Center of Reference and Excellence (UCARE), Institute of Allergology, Charité - Universitätsmedizin Berlin, Corporate Member of Freie Universität Berlin and Humboldt-Universität zu Berlin, Berlin, Germany. Fraunhofer Institute for Translational Medicine and Pharmacology ITMP, Immunology and Allergology, Berlin, Germany.	1
Inmaculada	Martinez-Saguer	Haemophilia Centre Rhine Main, Mörfelden-Walldorf, Germany.	2
Mathias	Sulk	Department of Dermatology, University of Münster, Münster, Germany.	2
Susanne	Trainotti	Department of Otorhinolaryngology, Head and Neck Surgery, Klinikum Rechts der Isar, TUM School of Medicine and Health, Department Clinical Medicine, Munich, Germany.	3
Bettina	Wedi	Department of Dermatology and Allergy, Comprehensive Allergy Center, Hannover Medical School, Hannover, Germany.	3

**Table 2.** Definition of consensus level by the expert group. The relative level of agreement underlying consensus strength categories is shown.

Definition of consensus level		
Level of consensus	Level of agreement	
Unanimous consensus	100% of participants	
Strong consensus	≥ 85% of participants	
Consensus	70 – 84% of participants	
No consensus	< 70% of participants	

**Table 3.** Keynotes of consensus groups regarding the long-term treatment of hereditary angioedema patients with lanadelumab. The proposed final 20 keynote statements numbered according to the treatment phase are shown.

**Phase of management**	**Keynote**
1 Introduction	Shared decision-making is already well established, with physician–patient communication conducted on an equal footing.
	Presenting all treatment options completely neutrally is challenging, as the treating team is aware of the patient’s history and preferences.
	Route and frequency of administration are pivotal decision criteria for patients.
	Neutral, comparative educational material covering all therapies can be helpful.
	Study patients are managed separately because additional effort is required to motivate them for trial participation.
2 Initiation	The Angioedema Control Test (AECT) and the Angioedema Quality of Life questionnaire (AE-QoL) are incorporated when determining treatment suitability.
	Ideally, the first injection should be administered on site; however, highly individualized; arrangements are possible (self-administration, family physician, or patient-support service).
	Treatment-planning considerations; provide information material and prescriptions for lanadelumab (2- or 6-vial packs), together with an initiation folder, emergency medication, and short-term prophylaxis (STP) instructions.
	Conduct a follow-up consultation after the second or third injection.
	Switching from other long-term prophylaxes (LTPs) to lanadelumab. For attenuated androgens, taper the dose and monitor for breakthrough attacks. For C1-INH, a single overlap dose with lanadelumab may be used, tailored to the patient. For oral berotralstat, limit overlap with lanadelumab to a maximum of 1 week.
3 Monitoring	Always consider the individual clinical situation, including current disease activity.
	Aim for complete absence of attacks (and prodromal symptoms); reassurance regarding attack control should be achieved no earlier than two to three months after the first injection.
	Patients should proactively report comorbidities or other relevant changes.
	Provide clear channels for patient-initiated contact between scheduled visits.
	Establish center infrastructure to handle incoming queries efficiently.
	Emphasize to patients the importance of regular follow-up visits.
	Take action if severe or frequent attacks occur, during family-planning or pregnancy, or if prescriptions are no longer filled.
	Investigate causes of suboptimal response (e.g., lifestyle factors, comorbidities) and consider a defined observation period before contemplating therapy modification.
	Introduce patient support programs or patient-advocacy groups as needed.
	Supply written information on STP and emergency medication, and offer appointment or prescription reminders.

**Table 4.** HAE Expert Consensus Symposium 2024 consensus statements for the long-term treatment of hereditary angioedema patients with lanadelumab. The final 10 consensus statements numbered according to the treatment phase (S1 and so on) are ordered by concept phase, which is summarized in Table 1. The level of agreement is shown.

**Consensus statements for the long-term treatment of HAE patients with lanadelumab**			
**Phase of management**	**Item**	**Statement**	**Strength of Consensus**
1 Introduction	1	The decision to initiate long-term prophylaxis is made through a shared decision-making process.	
	2	It is emphasized that the decision is not final and can be revised or adjusted at any time.	
	3	After an introductory presentation of the approved treatment options in terms of route of administration and dosing interval, the options under consideration are presented in detail, taking into account patient-specific requirements and patient preferences.	
2 Initiation	4	After appropriate training in injection technique, administration can be performed by the patient. All patients should be offered the first injection at the center.	
	5	Therapy is started with an injection interval of 2 weeks and then adjusted individually for each patient.	
	6	a) Emergency medication should continue to be available and carried even when there are no attacks. b) Peri-interventional prophylaxis should be considered in consultation with relevant specialists.	
3 Monitoring	7	In most patients, an extension of the injection interval is possible without loss of efficacy and can be offered to symptom-free patients.	
	8	Adjustment of the injection interval over time is subject to the primary therapeutic goal of complete disease control.	
	9	The patient should be encouraged to proactively report relevant new issues (e.g. co-morbidities, changes in lifestyle or disease activity) to the center. This does not detract from the importance of regular visits. This does not affect the relevance of regular visits.	
	10	Family planning and the desire to have children should be discussed and considered before and during treatment in women of childbearing age.	

## Supplemental material

Supplemental material Original consensus statements in German.
