# Supplementary material for: Expert consensus on the long-term use of lanadelumab in hereditary angioedema: Toward harmonized care
Source: Allergol Select. 2026 May 5;10:86–97. doi: 10.5414/ALX02622E (PMC13151902; doi:10.5414/ALX02622E)
Supplement: Supplemental material [file allergologieselect-10-086-S01.pdf]

1. Die Entscheidung für eine Langzeitprophylaxe wird per Shared Decision Making getroffen.

Zustimmung 7/7, 100%

2. Dabei wird betont, dass die Entscheidung für eine Langzeitprophylaxe nicht endgültig ist und jederzeit revidiert oder angepasst werden kann.

Zustimmung 7/7, 100%

3. Nach einleitender Darstellung der zugelassenen Therapieoptionen hinsichtlich Applikationsform und Verabreichungsintervall werden unter Berücksichtigung von patientenindividuellen Voraussetzungen und Patient*innenwünschen, die in Frage kommenden Optionen detailliert dargestellt.

Zustimmung 7/7, 100%

4. Nach entsprechender Schulung der Injektionstechnik kann die Verabreichung von Lanadelumab durch die Patient*innen durchgeführt werden. Allen Patient*innen soll die Durchführung der 1. Injektion am Zentrum angeboten werden.

Zustimmung 6/7, 85.7%

5. Die Therapie mit Lanadelumab wird bei jugendlichen und erwachsenen Patient*innen wird mit einem 2-wöchigen Injektionsintervall gestartet und im weiteren Verlauf patientenindividuell angepasst.

Zustimmung 7/7, 100%

6. a) Notfallmedikation muss auch bei Attackenfreiheit weiterhin vorgehalten und mitgeführt werden.

Zustimmung 7/7, 100%

6. b) Eine peri-interventionelle Prophylaxe soll in Absprache mit den jeweiligen Fachdisziplinen erwogen werden.

Zustimmung 7/7, 100%

7. Bei den meisten Patient*innen ist eine Verlängerung des Injektionsintervalls ohne Wirksamkeitsverlust möglich und kann symptomfreien Patient*innen angeboten werden.

Zustimmung 7/7, 100%

8. Die Anpassung des Injektionsintervalls im weiteren Verlauf unterliegt dem obersten Therapieziel der vollständigen Krankheitskontrolle.

Zustimmung 7/7, 100%

9. Der Patient*in soll angehalten werden, proaktiv relevante neue Aspekte (z.B. Komorbiditäten, Änderung der Lebenssituation / Krankheitsaktivität) an das Zentrum zu melden. Die Relevanz regelmäßiger Visiten bleibt dadurch unberührt.

Zustimmung 7/7, 100%

10. Bei Frauen im gebärfähigen Alter sollen Familienplanung und Kinderwunsch vor und während der Therapie besprochen und berücksichtigt werden.

Zustimmung 7/7, 100%
